# Unravelling the effect of experimental pain on the corticomotor system using transcranial magnetic stimulation and electroencephalography

**DOI:** 10.1007/s00221-017-4880-0

**Published:** 2017-02-10

**Authors:** Marylie Martel, Marie-Philippe Harvey, Francis Houde, Frédéric Balg, Philippe Goffaux, Guillaume Léonard

**Affiliations:** 1 Faculty of Medicine and Health Sciences, Université de Sherbrooke, Sherbrooke, QC J1H 5N4 Canada; 2 Research Centre on Aging, CIUSSS de l’Estrie-CHUS, 1036, rue Belvédère Sud, Sherbrooke, QC J1H 4C4 Canada; 3 Department of Surgery, Faculty of Medicine and Health Sciences, Université de Sherbrooke, Sherbrooke, QC J1H 5N4 Canada

**Keywords:** Pain, Experimental pain, Transcranial magnetic stimulation, Recruitment curves, Electroencephalography, Corticospinal tract, Motor cortex, Functional connectivity

## Abstract

The interaction between pain and the motor system is well-known, with past studies showing that pain can alter corticomotor excitability and have deleterious effects on motor learning. The aim of this study was to better understand the cortical mechanisms underlying the interaction between pain and the motor system. Experimental pain was induced on 19 young and healthy participants using capsaicin cream, applied on the middle volar part of the left forearm. The effect of pain on brain activity and on the corticomotor system was assessed with electroencephalography (EEG) and transcranial magnetic stimulation (TMS), respectively. Compared to baseline, resting state brain activity significantly increased after capsaicin application in the central cuneus (theta frequency), left dorsolateral prefrontal cortex (alpha frequency), and left cuneus and right insula (beta frequency). A pain-evoked increase in the right primary motor cortex (M1) activity was also observed (beta frequency), but only among participants who showed a reduction in corticospinal output (as depicted by TMS recruitment curves). These participants further showed greater beta M1-cuneus connectivity than the other participants. These findings indicate that pain-evoked increases in M1 beta power are intimately tied to changes in the corticospinal system, and provide evidence that beta M1-cuneus connectivity is related to the corticomotor alterations induced by pain. The differential pattern of response observed in our participants suggest that the effect of pain on the motor system is variable from on individual to another; an observation that could have important clinical implications for rehabilitation professionals working with pain patients.

## Introduction

Pain is a rapidly growing area of research, and the last years have shown huge advancement in our understanding of its neurophysiological process. The development of neuroimagery techniques have led to the discovery that pain perception is intimately linked to the activation of a complex cerebral network comprised, among other things, of the primary somatosensory cortex (S1) and the secondary somatosensory cortex (S2), the anterior cingulate cortex (ACC) and the insula (IC) (Apkarian et al. 2005; Forster and Handwerker 2014; Nakata et al. 2014).

A few neuroimagery studies have also reported an increase in the activity of the primary motor cortex (M1) in the presence of experimental pain (Apkarian et al. 2000; Tracey et al. 2000; Burns et al. 2016). Stancák et al. demonstrated, using electroencephalography (EEG), that the application of a short-lasting painful heat stimuli on the hand decreased the β activity of the sensorimotor cortex (Stancák et al. 2007). Given the inhibitory role that β waves have on the motor cortex (Pogosyan et al. 2009), the decrease in M1 β activity noted by Stancák and colleagues suggests that the presence of a brief nociceptive stimulus could prime the motor brain regions (reduced inhibition), possibly to facilitate motor withdrawal responses. As pointed out, the results obtained by Stancák and colleagues were obtained following the application of brief/escapable, nociceptive stimuli and it remains uncertain whether the same pattern of results would be obtained with longer/unavoidable nociceptive stimulations.

The observations made with neuroimagery techniques are consistent with the results of studies performed with transcranial magnetic stimulation (TMS). TMS studies have shown that experimental pain stimulation can alter the excitability of the corticomotor system (Farina et al. 2001; Valeriani et al. 2001). However, contrary to the study by Stancák et al. (that suggest a priming of the motor cortex in the presence of pain), TMS studies generally report reduced corticospinal excitability following nociceptive stimuli (Boudreau et al. 2007; Mercier and Leonard 2011; Schabrun and Hodges 2012; Schabrun et al. 2013; Rittig-Rasmussen et al. 2014). Some researchers have suggested that these corticomotor effects could explain the negative impact that pain can have on motor learning (Boudreau et al. 2007; Rittig-Rasmussen et al. 2014). Supporting this are the results of Rittig-Rasmussen et al. (Rittig-Rasmussen et al. 2014) who have observed that the change in corticospinal excitability (increased motor-evoked potential [MEP] amplitudes) noted following upper trapezius training was completely blocked by a hypertonic muscle saline injection, with the effect being apparent up to 7 days post-training.

Interestingly, several neuroimagery and neurostimulation studies have shown that patients suffering from clinical pain conditions show changes in cortical representation at the M1 level. For example, in patients suffering from complex regional pain syndrome (CRPS) and from phantom limb pain, researchers have reported reduced cortical representation of the affected limb (Karl et al. 2001; Krause et al. 2006). Although compelling, these studies remain correlational and it is impossible to know if the neuroplastic changes in M1 are directly caused by pain. The use of an experimental pain paradigm, in which the researchers can manipulate the presence of pain, would make it possible to address this question and determine whether pain is causally linked to corticomotor changes.

In this study, TMS and EEG were used concomitantly to better understand the effect of pain on the motor system. More specifically, the objectives were to evaluate the effect of a prolonged/inescapable nociceptive stimulation on TMS recruitment curves [a measure believed to reflect the strength of the corticospinal projections (Devanne et al. 1997; Abbruzzese and Trompetto 2002)] and on the pattern of EEG activity of the motor brain regions. A second objective was to determine if these potential changes in the TMS recruitment curve and EEG activity could be related to changes in functional connectivity between M1 and other brain regions implicated in the perception of pain.

## Materials and methods

### Participants

Nineteen healthy, right handed adults (12 women and 7 men; mean age 29 ± 7 years old) participated in the study. To be included in the study, participants had to be aged over 18 years and be pain-free (absence of painful health condition and no pain upon testing). For security reasons, individuals with neurological disorders, metal implants in the skull, a pacemaker or neurostimulator, epilepsy or pregnant were excluded from the study. Participants were asked to refrain from consuming caffeine for 6 h before testing, and tobacco products for 2 h before testing. The research protocol was approved by the ethics committee of the Research Centre on Aging (Sherbrooke, Quebec, Canada) and each participant provided informed written consent before participating in the study.

### Transcranial magnetic stimulation (TMS)

Magnetic stimuli were delivered by a 70 mm figure-eight coil connected to a Magstim 200 (Magstim Co., Dyfed, UK). Participants sat in a comfortable chair and two Ag/AgCl surface recording electrodes (1 cm^2^ recording area) were positioned over their left first dorsal interosseous (FDI) muscle to record motor-evoked potentials (MEP). Electromyographic signals, elicited by the magnetic stimuli, were amplified and filtered (bandwidth 200 Hz to 2 kHz) with a CED 1902 amplifier (Cambridge Electronic Design Limited, Cambridge, UK), and digitized at a sampling rate of 10 kHz using a Power 1401 mk II interface and Spike 2 software (version 7.10; Cambridge Electronic Design Limited, Cambridge, UK).

With the coil held ~45° in the mid-sagittal plane, the approximate location of the FDI muscle on the right hemisphere was explored in 1-cm step until reliable MEP could be evoked in the FDI. The optimal location for eliciting MEP in the FDI was found (hotspot). This site was then marked on the scalp of the participants with a marker to ensure consistent coil positioning. Throughout the experiment, the experimenter frequently reassessed the coil position to ensure that it remained over the optimal stimulation site. At this point, stimulations of varying intensities were sent to determine the resting motor threshold (rMT), defined for each participant as the minimal intensity of stimulation capable of eliciting MEPs of at least 50 µV in 50% of the trials with the FDI at rest (no muscle contraction). Then, 4 blocks of 10 stimulations were provided randomly to participants (delay between each stimulation = 5 to 8 s), with the stimulation in each block given at the same intensity (i.e., 90, 110, 130, and 150% of rMT). The peak-to-peak amplitude of MEP responses were measured off-line and averaged for each participant to derive mean values. The slope of the recruitment curve (describing the relationship between MEP amplitude and TMS intensity) was then calculated using hierarchical linear modeling (HLM) (Roberts et al. 2010).

### Electroencephalography (EEG)

EEG activity was recorded at rest using a 32-channel EEG acquisition system (Brain Products GmBh, Munich, Germany) with electrodes positioned according to the international 10–20 system. Data were recorded at 500 Hz for 5 min using FCz reference and keeping all electrode impedances below 5 kΩ. Eye blinks and motion artifacts were removed from the data using independent component analysis (ICA) denoising (Brain Vision Analyzer, Brain Products GmbH, Munich, Germany). Data were then re-referenced to the common average.

For each participant, 15 non-overlapping, 2-s segments without artifacts were randomly selected and decomposed in eight frequency bands: *δ* (delta: 1.5–4 Hz), *θ* (theta: 4–8 Hz), *α*1 (alpha 1: 8–10 Hz), *α*2 (alpha 2: 10–13 Hz), *β*1 (beta 1: 13–21 Hz), *β*2 (beta 2: 21–30 Hz), *β*3 (beta 3: 30–60 Hz) and *ω* (omega >60 Hz). For each segment, intracranial source current densities were then computed using sLORETA software (Pascual-Marqui 2002), yielding sources in 6239 5 × 5 × 5 mm^3^ cortical grey matter voxels in standard MNI space (Fonov et al. 2011). sLORETA allows the localization of spatially distributed sources of activity without *a priori* on their number, which is well suited in the context of pain (Apkarian et al. 2005; Tracey and Mantyh 2007; Schweinhardt and Bushnell 2010). Current density maps were then averaged across segments for each subject and condition (i.e., baseline and pain condition).

### Capsaicin application

**Fig. 1:**
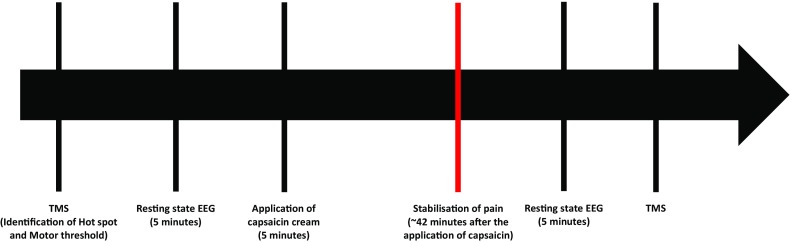
Timeline of the experimental procedures

After the evaluation of baseline TMS and EEG measures, experimental pain was induced by a 1% capsaicin cream. More specifically, 0.06 ml of capsaicin was applied on the middle volar part of the left forearm in a perimeter of 4 × 4 cm. Capsaicin-induced pain was evaluated by the participants using a visual analogue scale (VAS; 0 = “no pain”, 10 = “the worst imaginable pain”), every 5 min until the pain sensation stabilized (i.e., when participants rated same intensity of pain in 2 consecutive VAS pain measures). Once the pain became stable, EEG and TMS measures were assessed again (see Fig. 1).

### Statistical analysis

Paired-sample* t* tests were used to determine if there was a difference between the baseline and pain condition for the HLM values. Changes in current density power (EEG activity) between the baseline and pain condition were assessed using paired-sample t-tests across subjects, independently for each frequency band and each voxel. Statistical significance was assessed through statistical nonparametric mapping using 5000 randomizations to account for multiple comparisons. A threshold on the t-statistic corresponding to *p* < 0.05 was used to uncover pain-evoked activation maps and identify regions of the brain displaying changes in activity between the rest and pain conditions.

Because the analyses revealed no consistent changes in TMS measures and EEG activity between the baseline and pain condition (see Results section below), separate functional connectivity analyses were conducted in participants who showed a reduction in corticospinal output and an increase in M1 β activity (group 1), and in participants who did not (group 2). For each group, linear lagged connectivity was assessed in the β band frequency using sLORETA software between M1 (region of interest) and other brain regions in which an increase in activity was observed during the pain condition. These functional connectivity analyses allowed us to evaluate if the activation of M1 was related to an interaction with other brain structures also activated in the presence of pain (Apkarian et al. 2000; Tracey et al. 2000).

## Results

### Pain assessment

Every participant experienced pain following capsaicin application (mean pain intensity = 4 ± 2). On average, 42 min were required after capsaicin application before the pain stabilized.

### Effect of experimental pain on TMS recruitment curves

**Fig. 2:**
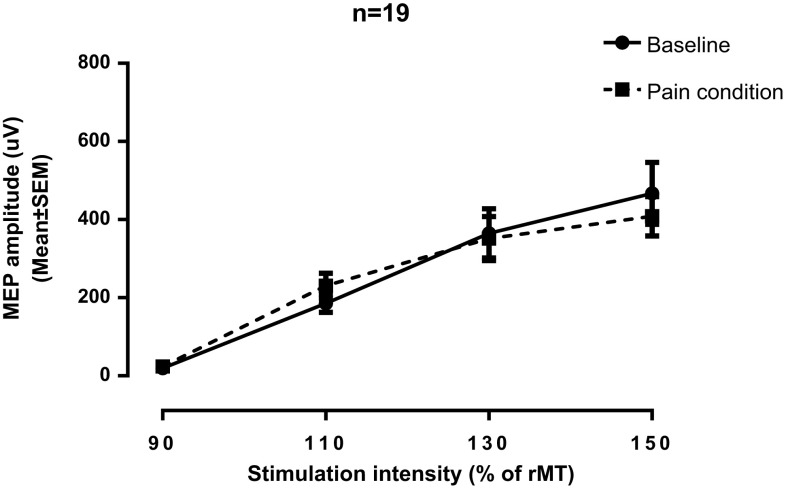
TMS recruitment curves obtained before and during pain. HLM analyses were used to estimate the slope of the recruitment curves of each participant. There was no change in the HLM slope value between the baseline and the pain condition in the total sample (*n* = 19), indicating that pain had no consistent effect on corticospinal output

TMS recruitment curves obtained before and after capsaicin application are presented in Fig. 2. As can be seen from this figure, pain did not affect corticospinal output, as evidenced by the comparable TMS recruitment curves obtained for the baseline and pain condition. The absence of difference between the two conditions was confirmed by the statistical analysis, with the paired-sample* t* test showing no difference in HLM slope values between the baseline and pain condition (*p* = 0.26). Pearson correlational analyses showed that there were no relationships between the change in the slope of the recruitment curve and the time needed for pain to reach a plateau (*r* = −0.02; *p* = 0.92) and between the change in the slope of the recruitment curve and the intensity of pain reported by the participants (*r* = −0.21; *p* = 0.36).

### Effect of experimental pain on brain activity

Source localization analyses conducted to compare brain responses between the baseline and pain condition revealed a significant increase in brain activity across the central cuneus (*x* = 0, *y* = −85, *z* = 10 at theta frequency), the left dorsolateral prefrontal cortex (DFPLC) (*x*= −45, *y* = 30, *z* = 35 at alpha frequency), and the left cuneus (*x* = −20, *y* = −90, *z* = 35) and right insula (*x* = 35, *y* = −5, *z* = 20 both at the beta frequency) while participants were in the pain condition (all ts >4.40, corresponding to *p* < 0.05). No changes were noted in other brain regions, including M1 (all *p* values >0.05).

### Between-group analyses

**Fig. 3:**
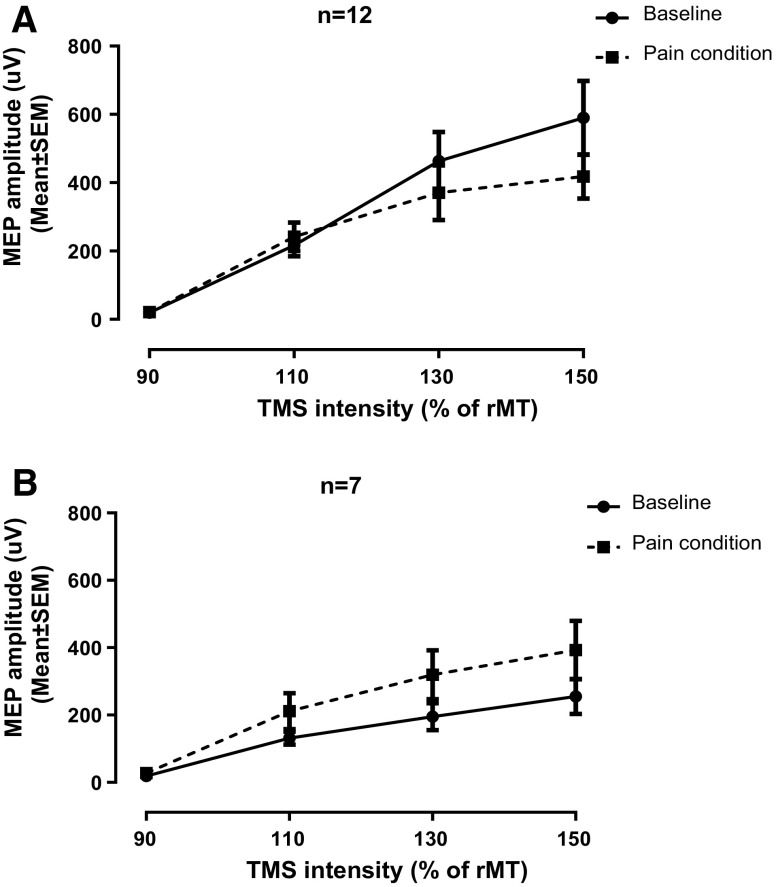
TMS recruitment curves obtained before and during pain. About two-thirds of the participants (*n* = 12) showed a decrease in corticospinal output during the pain condition (**a**), while the other third (*n* = 7) showed an increase in corticospinal output (**b**)

**Fig. 4:**
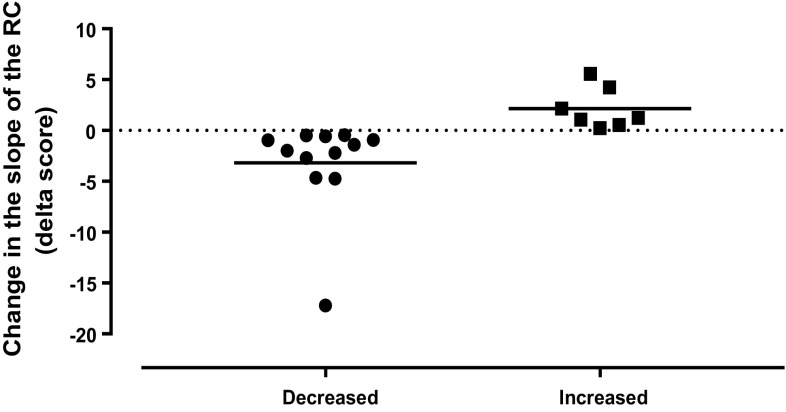
Delta scores, reflecting the change in the slope of the recruitment curves (RC). Delta scores were calculated for each participant by subtracting the HLM slope value obtained during the pain condition from the HLM slope value obtained during baseline condition. Negative delta scores indicate reduced recruitment curve slopes, while positive scores indicate an increase in the slope of the recruitment curve

Careful examination of the data revealed that about two-thirds of the participants (*n* = 12) showed a decrease in corticospinal output (reduced TMS recruitment curve slope) during the pain condition while the other third (*n* = 7) showed an increase in corticospinal output (increased TMS recruitment curve slope; see Figs. 3a, b, 4). These observations brought us to evaluate and to compare the changes in EEG brain activity and functional connectivity between these two groups of participants.

**Fig. 5:**
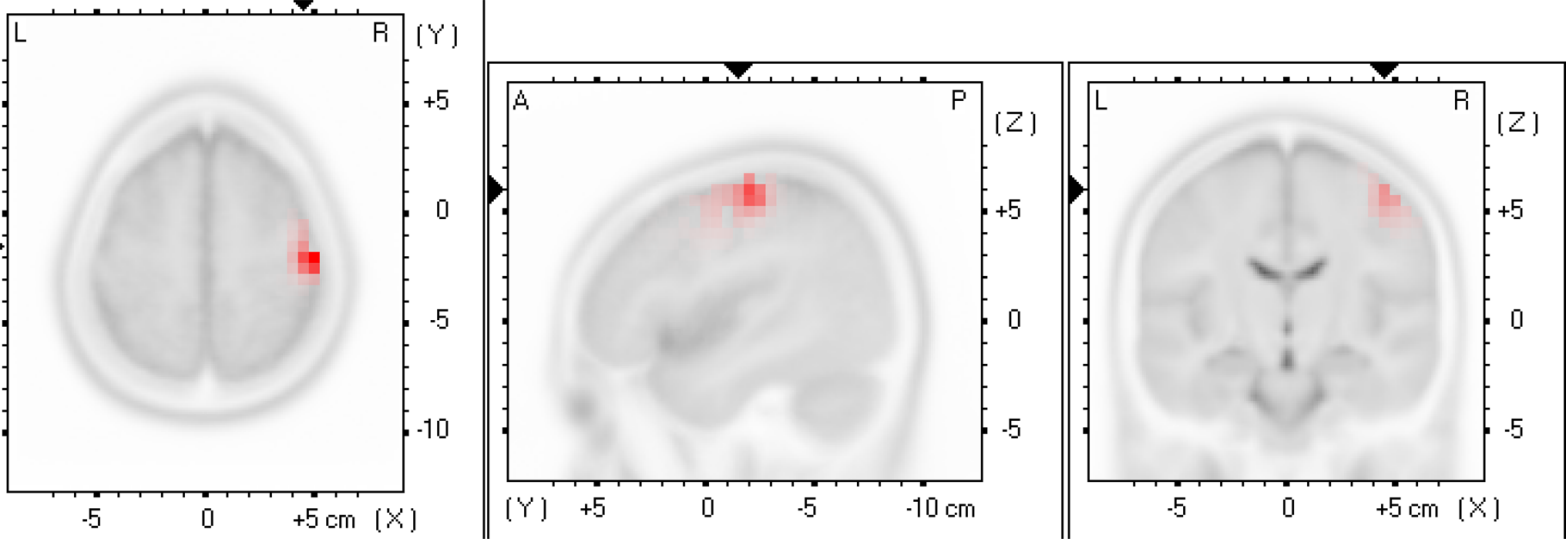
A pain-evoked increase in the right primary motor cortex activity during the pain condition was observed (beta frequency), but only among participants who showed a decrease in corticospinal output (*n* = 12)

The between-group analysis first revealed that, compared to participants who showed an increase in corticospinal output, participants who showed a decrease in corticospinal output also showed greater right M1 beta frequency activity (*x* = 35, *y* = −15, *z* = 50; *t* = 4.69, *p* = 0.049) in the pain condition (see Fig. 5). Importantly, this group difference was absent at baseline (all ts <4.80, *p* > 0.48). Between-group comparisons, looking at changes in EEG functional connectivity, showed that, compared to participants who showed an increase in corticospinal output, those who showed a decrease demonstrated greater pain-related beta M1-cuneus connectivity (*t* = 3.58, *p* = 0.03). Again, these between group differences in beta M1-cuneus connectivity were not found at baseline (*t* = 3.73, *p* = 0.73). No other connectivity change was observed (all *p* values >0.05).

## Discussion

The current study’s objective was to better understand the corticomotor changes induced by pain. More specifically, we wanted to determine if a prolonged/inescapable nociceptive stimulation pain, induced with a capsaicin cream, could modify TMS recruitment curves as well as EEG activity of the motor cortex, and if these eventual alterations could be associated to functional connectivity changes. Our analyses revealed that capsaicin pain produced variable effects, with approximately two-thirds of participants showing a reduced TMS recruitment curve slope. Participants who showed this type of decrease also showed an increase in M1 β activity.

### Effect of pain on cortical representation and corticospinal output

In the past years, many studies have revealed the presence of functional reorganizations in the somatosensory and motor system of pain patients. For example, Krause et al. observed that patients with complex regional pain syndrome (CRPS) had a smaller corticomotor representation of the affected limb, compared to pain-free participants (Krause et al. 2006). Flor et al. reported similar changes in the primary somatosensory cortex (S1) in people suffering from phantom pain (Flor 2003). Interestingly, researchers observed the presence of a positive correlation between pain intensity and the amplitude of cortical reorganization in amputee patients, suggesting that these neuroplastic changes could play an important role in the physiopathology of persistent pain (Flor et al. 1995).

The idea that cortical reorganization could play an important role in the physiopathology of chronic pain was reinforced by Maihofner et al. and Pleger et al. who observed a normalization of the cortical changes in CRPS patients after successful treatment, once pain subsided (Maihofner et al. 2004; Pleger et al. 2005). The results of Maihofner et al. and Pleger et al. support the idea that pain could drive cortical reorganization; however, the ultimate way to confirm the presence of a causal relationship between pain and cortical changes is to experimentally manipulate the presence of pain, as it is the case in this study. Our results show that pain can, indeed, drive changes in the corticomotor system, but that its effect is not uniform across all individuals.

Nevertheless, we must remember that the results obtained from experimental pain paradigm cannot be directly generalized to clinical pain populations. It should also be noted that the effect of pain on the motor system can vary depending on the duration of the painful stimulus (phasic *vs* tonic pain), the submodality (deep *vs* superficial pain), and the location (proximal *vs* distal pain) (Valeriani et al. 1999, 2001; Farina et al. 2001; Le Pera et al. 2001; Cheong et al. 2003; Svensson et al. 2003; Mercier and Leonard 2011). Replicating the present results with different experimental pain paradigms and pursuing research in pain populations is essential before any final conclusions can be made.

### Effect of pain on EEG activity of the motor cortex

Several neuroimaging studies have shown that experimental pain can affect the activity of the motor cortex (Apkarian et al. 2000; Tracey et al. 2000; Burns et al. 2016). For the most part, these studies were done using functional magnetic resonance imaging (fMRI). Although useful—in particular because of its ability to measure changes in deep areas of the brain—it is important to remember that fMRI BOLD responses reflect changes in cerebral blood flow, cerebral blood volume and cerebral metabolic rate of oxygen following neural activation (Fox and Raichle 1986; Uludag et al. 2009; Attwell et al. 2010). As such, changes in BOLD can, at best, be related to changes in neural activity and cannot be interpreted specifically in terms of excitatory (increase in the activity of excitatory neurons) or inhibitory (increase in the activity of inhibitory neurons) activity. Contrary to fMRI, EEG directly measures the neuroelectric activity of brain cells, allowing a better characterization of neuronal changes (Aine 1995). In this study, the EEG analyses have revealed that the majority of participants showed increased contralateral M1 β frequency activity during pain, suggesting that nociception increases the inhibitory activity in this area (Pogosyan et al. 2009). The biological reasons for these cortical changes remain hypothetical. A possible explanation is that increased β activity could force the injured individual to limit his movements, to promote healing. However, in certain cases, this inhibitory effect could be detrimental, for example by interfering with motor learning and rehabilitation (Boudreau et al. 2007; Bouffard et al. 2014).

In the past years, accumulating evidence stemming from paired-pulse TMS studies has suggested that chronic pain populations display changes in GABA-mediated intracortical inhibition (see for instance Parker et al. (2016) for a review). Perhaps the most compelling observations are the ones made by Lefaucheur and colleagues (Lefaucheur et al. 2006). In this study, Lefaucheur and colleagues observed that (1) neuropathic pain patients had reduced intracortical inhibition, when compared to age-matched healthy controls, (2) application of high-frequency (10 Hz) repetitive TMS (rTMS) in these pain patients increased intracortical inhibition, and (3) there was a significant association between the extent of pain relief and the increase in intracortical inhibition observed following the application of rTMS. Changes in GABA-mediated short intracortical inhibition (SICI) have also been documented with experimental pain paradigms (Fierro et al. 2010; Schabrun and Hodges 2012). Results from these studies indicate that the effect of experimental pain on SICI may depend on the nature/location of the nociceptive stimulus; while Fierro et al. (2010) observed reduced SICI following a topical capsaicin application (superficial cutaneous pain), Schabrun and Hodges (2012) reported increased SICI following injection of a hypertonic saline solution (deep muscle pain). Changes in intracortical facilitation (ICF) were also noted by Schabrun and Hodges (2012), but not by Fiero et al. (2010). These findings help to better understand the role played by intracortical circuits and remind researchers that the effect of pain on the corticomotor system likely varies depending on the type of pain.

The increase in β power observed in the majority of our participants contrast with the results of Stancák and colleagues, who showed that thermode induced pain *decreased* M1 β activity (Stancák et al. 2007). This discrepancy could be explained by the fact that prolonged pain (e.g. capsaicin) and brief pain (e.g. thermode) stimulation may foster the emergence of different motor strategies. Whereas immobilization can be a successful strategy in the former case, this same response could be detrimental in the second case, when it is possible for the individual to remove the body part away from the painful stimuli. Decreasing β activity during brief/escapable nociceptive stimulation could promote movement and help the individual avoid potential threats.

Associations between M1 β power and GABA concentration have been observed by Baumgarten and colleagues (2016). Similarly, Farzan et al. (2013) noted that the duration of the silent period [a TMS measure mediated by GABA receptors (Abbruzzese and Trompetto 2002; Jono et al. 2016)] is related to β oscillations. Taken together, these observations suggest that the changes observed in corticospinal output in some of our participants could be linked to changes in GABA activity.

### Effect of pain on other brain areas

The EEG analysis revealed an increase of the activity of the insula, DFPLC and cuneus in the pain condition in all participants, when compared to baseline. The role of the insula and DFPLC in pain perception and modulation has been well documented in previous pain studies (Rainville et al. 2000; Borckardt et al. 2007); however, the activation of the cuneus in the pain condition is more unexpected. A previous study, from our research group, did suggest that a brain area adjacent to the cuneus could play a significant role in the perception of pain (Goffaux et al. 2014). In this past study, we observed that individuals who showed increased activity in the precuneus in the presence of experimental pain also showed the promptest response to pain. Traditionally linked to the treatment of visual information (Corbetta et al. 1995; Nobre et al. 2003), the cuneus also plays an important role in the integration of sensory information, as well as cognitive processes such as attention, learning and memory (Cabeza et al. 2002; Makino et al. 2004).

The functional connectivity analyzes, done on the subgroup of participants for whom pain reduced corticospinal output, further highlight the potential role that the cuneus could play in pain processes. These analyses have shown that the application of a capsaicin cream increases the functional connectivity between the motor cortex and the cuneus in individuals who show a reduced TMS recruitment curve slope. These results reinforce the role that the cuneus could play as a significant brain area for the integration of sensory and attentional information. This integrative function of the cuneus makes it an ideal cerebral structure, capable of modulating the activity and organization of the motor cortex based on the ascending sensory information and on the context in which the individual is placed and asked to interact.

## Limits

The most important limit of this study probably relates to the inconsistent effect produced by pain on the corticomotor system. Indeed, it should be reminded that the most compelling findings (i.e., increased M1 β activity and reduced corticospinal output) were found in a subsample of participants. Future studies need to be conducted to determine if these results can be consistently reproduced and validate that the observed TMS and EEG changes are not spurious effects only. An additional limitation concerns the absence of control group. Although the TMS and EEG measures have been proven to be reliable (Cacchio et al. 2009; Cannon et al. 2012; Ngomo et al. 2012), the addition of a control group would have been an important asset for the study to document the stability of the TMS and EEG measures over time. Finally, it should be noted that the effect of pain on TMS and EEG measures was investigated only once (i.e., when pain stabilized). Again, futures studies, looking into the long-term effects are warranted.

## Conclusion

In conclusion, our results show that tonic experimental pain increases M1 β activity in certain individuals, and that this increase in β activity is intimately tied to corticomotor and functional connectivity changes. These observations remind us that the cerebrum works as an integrated system of circuits and that certain brain areas, other than those classically involved in pain perception and modulation can be affected by nociceptive stimulations. The differential pattern of response observed in our participants suggest that the effect of pain on the motor system is variable from an individual to another; an observation that could have important clinical implications for rehabilitation professionals working with pain patients.
